# Ceftaroline Fosamil as an Alternative for a Severe Methicillin-resistant Staphylococcus aureus Infection: A Case Report

**DOI:** 10.7759/cureus.3776

**Published:** 2018-12-25

**Authors:** Talha N Jilani, Syed O Masood

**Affiliations:** 1 Internal Medicine, Ziauddin University, Karachi, PAK; 2 Infectious Diseases, University of Cincinnati Medical Center, Cincinnati, USA

**Keywords:** methicillin-resistant staphylococcus aureus (mrsa), ceftaroline fosamil, mrsa

## Abstract

Bacteremia secondary to methicillin-resistant Staphylococcus aureus (MRSA) is a dreaded medical condition that is not only associated with a significant medical cost but also carries high morbidity and mortality. The poor clinical outcomes seen in MRSA patients and the nephrotoxic effects of high-doses of vancomycin are challenging its current status as the first-line treatment for MRSA. Fortunately, vancomycin-intermediate-staphylococcus aureus (VISA) and vancomycin-resistant-staphylococcus aureus (VRSA) are not common in the United States. However, MRSA still presents different treatment challenges. Elevated vancomycin minimum inhibitory concentrations (MICs) commonly result in decreased efficacy and an increased probability of treatment failure, prompting the use of alternative agents. Although daptomycin is an alternative, adverse effects (i.e., elevations in serum creatine phosphokinase (CPK), drug-induced myopathy, peripheral neuropathy, and eosinophilic pneumonia) may limit its use in some patients. In the search for a suitable replacement for vancomycin, great promise has been shown by anti-MRSA cephalosporins.

We present a case of MRSA bacteremia and endocarditis requiring a different approach to treatment as compared to traditional treatment with vancomycin alone. This case report describes the successful treatment of MRSA bacteremia with ceftaroline fosamil in a patient who responded poorly to conventional therapy, specifically vancomycin, due to an elevated MIC (2 µg/mL).

## Introduction

Staphylococcus aureus (S. aureus) is the bacterial microorganism known to be the second most common cause of bloodstream infections and is frequently responsible for high morbidity and mortality [1-2]. According to several population-based research studies performed over the last decade, it has an incidence rate of 15 to 40 episodes per 100,000 inhabitants per year and a mortality rate of around 15%-25% [1-3]. Compared to patients infected with methicillin-susceptible Staphylococcus aureus (MSSA) bacteremia, patients with methicillin-resistant Staphylococcus aureus (MRSA) had an increased risk of mortality and this directly correlates to a significant increase in direct medical costs and prolonged hospital stays [4-5].

Bacteremia and infective endocarditis (IE) caused by MRSA have become increasingly difficult to treat over the past decade, with surprising suboptimal response rates of less than 50% [6]. Although daptomycin and vancomycin are the standard therapeutic options, treatment failures with either or both agents have become increasingly common [7]. The efficacy of new antibiotics in terms of reducing mortality in patients infected with S. aureus, especially MRSA, has not currently been verified.

We present a case of MRSA bacteremia and endocarditis, which showed limited response to both vancomycin and daptomycin, but the patient’s condition improved significantly with the use of ceftaroline fosamil (a fifth-generation cephalosporin). This antimicrobial is not in the current guidelines for infections caused by MRSA but has shown to be effective when used against MRSA infections [8].

## Case presentation

A 34-year-old female with an unremarkable past medical history presented to the hospital with an eight-day history of abdominal pain, nausea, and vomiting. She had reported chills, but there was no documented fever, and she was admitted to the medical floor. Her social medical history was significant for intravenous heroin use. On the medical floor, she developed respiratory decompensation and was moved to the intensive care unit (ICU). She started spiking high-grade fevers. She was empirically started on cefepime and vancomycin. Chest computed tomography (CT) was performed and showed septic pulmonary emboli. Soon afterward, she had to be intubated for hypoxic respiratory failure. Blood cultures drawn earlier came back positive for MRSA. The sputum culture also came back positive for MRSA. Antibiotics were de-escalated to vancomycin only. A two-dimensional (2D) transthoracic echocardiogram showed pulmonic valve endocarditis, pulmonic valve insufficiency, and tricuspid regurgitation.

Despite the maintenance of a vancomycin trough between 15 and 20 mcg/dl, her blood cultures remained persistently positive for MRSA. Initial blood cultures showed a vancomycin MIC of 1.0. After around 10 days of persistently positive blood cultures, vancomycin MIC increased to 2.0. At this time, for the treatment of MRSA bacteremia, we initiated daptomycin 10 mg/kg and continued vancomycin for MRSA pneumonia. Blood cultures remained positive despite the addition of daptomycin 10 mg/kg. After a discussion with the critical-care team, we decided to discontinue the current antibiotics and initiated ceftaroline fosamil 600 mg intravenous (IV) Q8 hour. Since Q12 hour dosing is approved for skin and soft tissue infections, we used Q8 hour dosing to address the more severe infection of endocarditis, persistent positive bacteremia, and MRSA pneumonia. After two additional days of ceftaroline fosamil use, her blood culture became negative. She spent another two days in the ICU and then was transferred to the step-down unit. We were able to send her to a nursing home to finish a six-week course of IV antibiotics with ceftaroline fosamil.

## Discussion

Ceftaroline fosamil is classified as a fifth-generation cephalosporin. Currently, it is approved by the Food and Drug Administration (FDA) for acute bacterial skin and skin structure infection (ABSSSI) and community-acquired bacterial pneumonia (CABP). The cephalosporins are known for their extensive spectrum of activity, relative efficacy, and favorable safety profiles [9]. Ceftaroline fosamil is effective against the gram-positive organisms (Streptococcus pneumoniae, Staphylococcus aureus, and Streptococcus pyogenes), and gram-negative species (Haemophilus influenzae and Moraxella catarrhalis), including resistant phenotypes [9].

By modifying the structure of the cephalosporin cefozopran, ceftaroline was created [10]. The pro-drug ceftaroline fosamil is rapidly transformed in the plasma to the bioactive agent ceftaroline with the help of the phosphono group, which increases its water solubility [11]. The 1,3-thiazole ring attached to the 3-position of the cephalosporin nucleus and the oxime group in the C7 acyl moiety is responsible for the enhanced anti-MRSA activity observed with ceftaroline. Ceftaroline binds to penicillin-binding protein mediating bactericidal activity. As per data based on multistep resistance selection studies, resistance to ceftaroline is relatively limited [12].

Successful treatment of a serious infection requires a triad of controlling the source of infection, an active immune system, and an effective antimicrobial agent. The patient presented in this article had a severe bloodstream infection involving multiple organs, and her blood culture remained persistently positive. In this patient’s circumstances, the issue was source control, as she had pulmonic valve endocarditis and septic pulmonary emboli. The surgical team assessed the patient and decided she was not stable for surgery. Repeat blood cultures over a 10-day duration showed an increase in vancomycin MIC from 1.0 to 2.0. Subsequent culture also showed a MIC of 2.0. This increase in MIC compromised the second component of the treatment triad, i.e., effective antibiotics for the successful treatment of bacteremia. Hence, we needed to switch antibiotics.

Our initial choice was to use two antibiotics, daptomycin and vancomycin. Daptomycin for bacteremia, as we were seeing an increased MIC in blood culture and continuing using vancomycin for pneumonia from septic emboli. MRSA causing septic pulmonary emboli and pneumonia could have been a heterogeneous strain as well. With previous reports of failure associated with vancomycin above 1.0 [13-14], we decided to switch over to ceftaroline fosamil.

Ceftaroline fosamil is currently approved for skin and soft tissue infections at a dose of 600 mg intravenous (IV) BID. In order to treat bacteremia involving multiple vital organs, we decided to use a higher dose of ceftaroline fosamil at 600 mg IV Q8 hours [15]. We were able to achieve clearance from bacteremia within 48 hours of starting ceftaroline fosamil. She was transferred out of ICU to the step-down unit. The patient continued to tolerate Q8 hour dosing without observed side-effects related to higher doses of ceftaroline fosamil [16]. It is not possible to say if it was a failure of vancomycin or daptomycin. If ceftaroline fosamil was started on day one on its own, it may have also taken 10-12 days to clear bacteremia instead of 48 hours. One can also argue that the 10-day use of vancomycin and the addition of daptomycin before switching to ceftaroline fosamil may have made it easier for ceftaroline fosamil to clear the bacteremia. Randomized controlled trials are needed to answer this question and to establish guidelines for the correct dosing of ceftaroline fosamil in the treatment of endocarditis.

In patients with MRSA bacteremia, with the involvement of the lungs and bacteremia with higher MIC, where the use of vancomycin becomes difficult, ceftaroline fosamil appears to be an efficient alternative antibiotic. The use of more than one antibiotic requires an increase in resources such as more than one line, more frequent dosing, assessment of the side-effect profiles of each antibiotic, and more drug interactions. It also results in a higher health care cost. Achieving a higher serum trough concentration is difficult, as vancomycin at a higher serum trough concentration of 15-20 mcg/ml increases the risk of renal failure [17]. Daptomycin is associated with rhabdomyolysis and thus requires the weekly monitoring of CPK. In these situations, ceftaroline fosamil is helpful, as it will require fewer resources and decrease the total amount of blood work required for safety monitoring. The use of a single antibiotic also decreases the resources and expenditure in terms of the number of peripherally inserted central catheter (PICC) lines, lumens, nurses providing care at the bedside and monitoring drips, and so on.

## Conclusions

Ceftaroline fosamil appears to be a good alternative for severe MRSA infection when standard antibiotics, such as vancomycin or daptomycin, cannot be used or are ineffective. However, more research is needed to identify a safe dosing schedule, readmission rate, and mortality rate before it can be approved for regular use.
